# Retrograde amnesia abolishes the self-reference effect in anterograde memory

**DOI:** 10.1007/s00221-023-06661-2

**Published:** 2023-07-14

**Authors:** Debora Stendardi, Flavia De Luca, Silvia Gambino, Elisa Ciaramelli

**Affiliations:** 1 Dipartimento di Psicologia, Università di Bologna, Bologna, Italy; 2 Centro Studi e Ricerche in Neuroscienze Cognitive, Cesena, Italy; 3 Present Address: School of Psychology, University of Sussex, Falmer, BN1 9QH UK

**Keywords:** Self, Self-reference effect, Focal retrograde amnesia

## Abstract

Is retrograde amnesia associated with an ability to know who we are and imagine what we will be like in the future? To answer this question, we had S.G., a patient with focal retrograde amnesia following hypoxia, two brain-damaged (control) patients with no retrograde memory deficits, and healthy controls judge whether each of a series of trait adjectives was descriptive of their present self, future self, another person, and that person in the future, and later recognize studied traits among distractors. Healthy controls and control patients were more accurate in recognizing self-related compared to other-related traits, a phenomenon known as the self-reference effect (SRE). This held for both present and future self-views. By contrast, no evidence of (present or future) SRE was observed in SG, who concomitantly showed reduced certainty about his personality traits. These findings indicate that retrograde amnesia can weaken the self-schema and preclude its instantiation during self-related processing.

In memory of Francesca Frassinetti

## Introduction

Philosophers and psychologists have long recognized the strong link between our ability to recollect past experiences and our sense of self-continuity over time, or self-sameness (Erikson 1968), which is at the basis of identity (Klein and Nichols 2012). Locke (1960/1894) held the extreme view that “someone who does remember nothing of his or her past literally has no identity” (p. 71). Modern cognitive psychology and neuroscience hold instead a more nuanced view that aims to understand which memory systems contribute to self-knowledge and one’s sense of self.

One interesting question in this respect is what remains of personal identity and the sense of self after severe retrograde memory loss, such as is observed in focal retrograde amnesia (FRA; De Renzi et al. 1997), a state of severely impaired memory for information acquired prior the onset of brain damage with normal or minimally impaired learning of new information (De Renzi et al. 1997; Levine et al. 1998). FRA is typically associated with multifocal lesions following traumatic brain injury (Markowitsch et al. 1993; Hunkin et al. 1995; Kapur 1999), herpes simplex encephalitis (O’Connor et al. 1992; Yoneda et al. 1994), and hypoxia (De Renzi and Lucchelli 1993; Reed et al. 1999), but it may also follow a (minor) physical injury, at times associated with psychogenic factors (Stracciari et al. 2005; Piolino et al. 2005; Arzy et al. 2011).

The retrograde memory deficit in FRA can encompass the entire lifespan (Levine et al. 1998; Ross 2000; O’Connor et al. 1992; De Luca et al. 2018), and most frequently consists of severely impaired autobiographical episodic memory with relatively preserved semantic knowledge (Dalla Barba et al. 1997; Levine et al. 1998; Manning 2002; Hunkin et al. 1995; Stracciari et al. 1994). As for personal semantics, usually FRA entails an initial loss of personal identity, but this is transient (Harrison et al. 2017). Indeed, since FRA patients have relatively preserved anterograde memory, they can easily re-learn several facts about themselves relatively soon. This usually means they reacquire autobiographical facts, such as where they were born, what was their job, who are their family members, while they remain incapable to re-experience past events, or feel emotionally detached from them, as if they were not part of their own past (Goldberg et al. 1981; Kapur et al. 1992, 1996; De Renzi and Lucchelli 1993; De Renzi et al. 1995). An FRA case we have recently described, S.G., makes a paradigmatic example (De Luca et al. 2018). After an initial phase in which he was unable to recall any personal event from his entire life, he quickly re-learned that he was married and had two daughters, as well as several facts about basketball, his lifelong passion. He remained, however, unable to remember specific events related to his family or basketball. Moreover, when questioned about basketball rules, SG showed full knowledge but no sign of personal involvement, as if he had nothing to do with basketball. He would describe his condition as "being born again, a new me" (De Luca et al. 2018).

Even though in FRA anterograde memory is—by definition—retained or minimally impaired, FRA patients’ anterograde memory performance may at times betray a weakened sense of self as well. Levine et al. (1998), for example, assesses anterograde memory in an FRA case, M.L., with a Remember/Know recognition memory task, which allows distinguishing retrieval with and without autonoetic consciousness. The results demonstrated that M.L. could use non-episodic processes to distinguish familiar from novel items (K responses), but he could not episodically re-experience post-injury events to the same extent as control subjects (R responses), as if he failed to complement recognition performance with self-related (autonoetic) information. ML had damage to the ventral frontal lobe and the uncinate fasciculus, a frontotemporal band of fibres previously hypothesized to mediate retrieval of specific events from one’s personal past (Markowitsch 1995). Piolino et al. (2005), too, found a deterioration of R responses (in retrograde memory) in an FRA case who had sustained a mild brain injury compared to matched controls, reflecting, again, an abnormal sense of self in memory.

Other evidence suggests, instead, a relative independence between self-knowledge and autobiographical memory. Conway and colleagues noted that self-knowledge fundamentally draws from abstract (semantic) representations, with time become detached from episodic memories (Conway and Rubin 1993). Accordingly, studies from Klein et al. (Klein and Loftus 1993; Klein et al. 2002a, b) have shown that the previous recollection of episodic memories does not influence reaction times when subjects decide whether a trait adjective is self-descriptive or not (and vice versa). Neuropsychological evidence also suggests some degree of functional independence: K.C. (Tulving 1993) and other amnesic cases (e.g., Klein et al. 1996; Klein et al. 2002a, b, c; Rathbone et al. 2009), who had lost most autobiographical memories, proved nonetheless capable to describe their personality accurately and reliably.

Assessing self-related processing in an FRA case represent a unique opportunity to test the effect of retrograde amnesia on the self-schema. Here, we assessed self-related processing in SG studying the self-reference effect (SRE; Rogers et al. 1977), a robust phenomenon observed in anterograde (recognition) memory indicating superior memory for items (e.g., personality traits) processed with respect to the self (e.g., “are you an introvert person?”) compared to another individual (e.g., “is she an introvert person?”; Symons and Johnson 1997; Klein and Kihlstrom 1986). The SRE is thought to be supported by regions in the default mode network, especially the medial prefrontal cortex (mPFC; Craik et al. 1999; Kelley et al. 2002; Davey et al. 2016), whose activity during self-trait judgments predicts which traits would be subsequently remembered (Macrae et al. 2004). Accordingly, patients with mPFC damage do not show the SRE (Philippi et al. 2012; Stendardi et al. 2021). The SRE has been attributed to the fact that in the self-referenced condition, incoming (trait) information is evaluated against the self-schema, a rich and articulated set of beliefs about oneself, generally deriving from the repeated categorization and subsequent evaluation of one’s behavior and experiences (Markus 1977). Importantly, we not only have a view of how we are now (present self-schema); we also anticipate how we will be like in the future (future self-schema; Libby and Eibach 2002; Pronin and Ross 2006). Indeed, healthy individuals also exhibit an SRE for items processed with respect to the future self (“will you be an introvert person ten years from now?”) compared to that of another individual (Stendardi et al. 2021).

To test the present and future SRE in SG, we asked him to judge whether each of a series of trait adjectives was descriptive of his present self, future self, another person, and that person in the future, and then recognize studied self-related and other-related present and future traits among distractors. Moreover, to begin to shed light on the mechanisms underlying SG’s SRE (or lack of), we asked him to judge, for each trait, how certain he was to possess (or not possess) that trait, and how important it was for him to possess that trait, separately for the present and future self. The fact that SG describes himself as ‘a new me’, has lost most of his past personal experiences, and recounts the few he still has as someone else’s, would lead to predict a reduced SRE in SG. Indeed, personal memories are a powerful source of information on our behavior, hence a major source of input to the self-schema. In order to ascribe the lack of SRE in SG to his retrograde memory impairment, and not, for example, to memory encoding deficits due to brain damage, we contrasted SG’s performance to that of two patients (AT and MR) with neurological memory disorders who had weak anterograde but normal retrograde memory, and with age-matched heathy controls. If, as we predict, retrograde memory plays a crucial role in imparting a preferential status to self-related information in memory, then the SRE should be reduced in SG but preserved in brain damaged patients with weak anterograde memory but preserved retrograde memory. Whether or not the purported reduction in the SRE in SG also extends to the future self will inform on the relation between retrograde memory and future-based cognition and qualify the clinical presentation of FRA.

## Materials and methods

### Participants

*Case SG.* SG has been described extensively in De Luca et al. (2018). For convenience, we reprise the details of his cognitive profile (see Table 1 for SG’s results in standardized neuropsychological tests) and the most important aspect of his clinical presentation. Our main interest here is in the new data relating to the SRE.

**Table 1:** Patients’ scores on standardized neuropsychological tests

	SG	AT	MR
MMSE	27/30	27/30	25/30
RPM	28.5 (3)	30.25 (4)	25 (2)
Verbal Judgement test	42.5 (2)	45 (2)	44.5 (2)
Attentional matrices	49.25 (4)	54.25 (4)	44.75 (3)
Stroop test			
RTs (s; cut-off = 27.5)	16.5	15.5	23.5
Errors (cut-off = 7.5)	0	0	5
TAP, alertness			
RTs (ms)	203 (4)	172 (4)	272 (4)
Errors	1	2	2
TAP, divided attention			
RTs (ms)	716* (1)	733 (3)	809 (0)**
Omissions	3 (4)	3 (4)	9 (0)**
TAP, Go Nogo			
RTs (ms)	575 (3)	455 (4)	714.5 (0)**
False reactions	2 (4)	1 (4)	0 (4)
TAP, Working memory			
RTs (ms)	597 (4)	633 (3)	1236 (0)**
Omissions	2* (1)	6 (1)*	9 (0)**
Verbal Fluency Task			
Semantic	44 (4)	62 (4)	41 (4)
Phonemic	46 (4)	48 (4)	26 (2)
Tower of London			
Total move score	17 (3)	39 (2)	27 (3)
Rules violation	0 (2)	1* (1)	1* (1)
Execution time (s)	104 (3)	295 (2)	283 (2)
Digit span	5.5 (4)	5.25 (4)	6 (4)
Corsi tapping test	5 (3)	6 (4)	3.75* (1)
Prose recall	8.10 (2)	12.5 (3)	11.5 (2)

The table reports corrected scores in standardized neuropsychological tests

*MMSE* Mini Mental State Examination, *RPM* Raven Progressive Matrices, *TAP* Test Battery for Attentional Performance. In parenthesis we report the Equivalent Scores (ES), ranging from 0 to 4, with 0 = impaired performance, 1 = borderline performance, and 2–4 = average to above average performance according to normative data (Spinnler and Tognoni 1987). Note that for the Alertness Subtest of TAP, there is a single ES indexing both RTs and omissions. Impaired performance is highlighted by a (**) and borderline performance is highlighted by a (*)

**Fig. 1:**
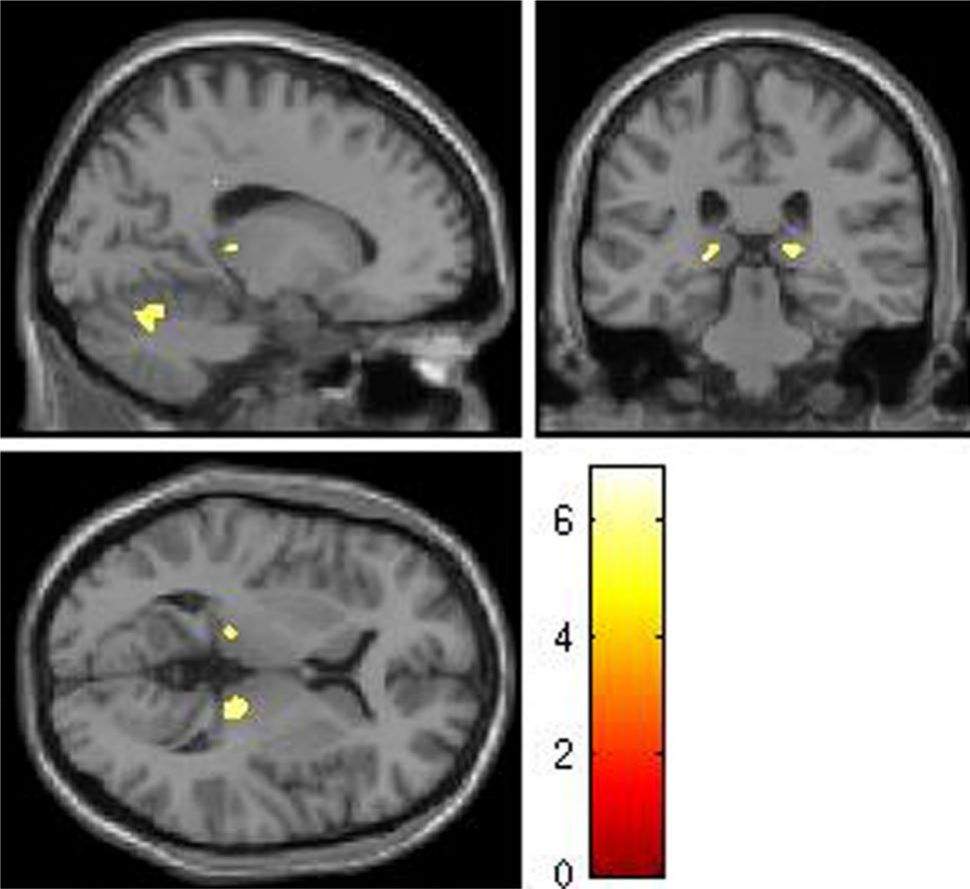
Regions of decreased grey matter volume in patient SG (*p* < 0.05, FWE-corrected). Clusters are superimposed on the SPM12 template

SG is a 50-year-old right-handed man with 10 years of education who worked as a painter. He was recruited at the Centro studi e ricerche di Neuroscienze Cognitive, Cesena, Italy. SG had an unremarkable medical history until June 2010, when, at the age of 43, he reported a myocardial infarction, followed by cardiac arrest and loss of consciousness (Glasgow Coma Scale = 4). Structural magnetic resonance imaging (MRI) revealed a perisylvian ventricular expansion, but no signal changes in the brain parenchyma and the structures of the posterior fossa. Voxel Based Morphometry (VBM;Ashburner and Friston 2000; Mechelli et al. 2005) detected significant areas of reduced grey matter volume in the fusiform gyrus, thalamus, and cerebellum (FWE-corrected threshold of *p* < 0.05), and, at a reduced threshold (*p* < 0.001, uncorrected), in the hippocampus, the anterior thalamus, and several regions of the autobiographical memory network, including the angular and supramarginal gyrus bilaterally (see Fig. 1).

Immediately after the brain insult, SG was unable to recall any personal event from his entire life, from childhood to the present days, including highly relevant events, such as the first date with his wife and the birth of their daughters. SG had lost personal semantic information as well: he was unable to report on what was his work was, where he lived. As anticipated, with time he could re-learn several aspects of his personal history, but he remained unable to remember specific past events. At the Autobiographical Interview (Levine et al. 2002), SG was completely unable to recall any event from the 11–17, 18–34, 35-last year life periods. He reported only two salient negative past events, one for the childhood decade (when his brother was born dead) and one for the last year (when he had a car accident). For the childhood event, SG reported fewer internal (episodic) details but a similar number of external details than controls, whereas his performance with the last year event fell within the normal limits (De Luca et al. 2018). SG showed a preserved ability to recognize famous faces and names at the Famous Face and Name Test (Rizzo et al. 2002) and in the ‘incidental knowledge’ section of the General Knowledge of the World Test (Mariani et al. 2002). He showed, however, impaired performance in the ‘encyclopaedic knowledge’ section of the General Knowledge of the World Test (including history, science, politics, arts), and in two additional tests assessing knowledge for famous remote events: the Italian questionnaire for remote events (Budriesi et al. 2002) and the Media Mediated Memory Test (Bizzozero et al. 2004).

S.G.’s performance was contrasted with that of 10 healthy controls (all males) matched to SG for age and education (mean age = 51, range = 44–56, mean education = 12.5, range = 8–18, *p* > 0.23 in both cases).

**Fig. 2:**
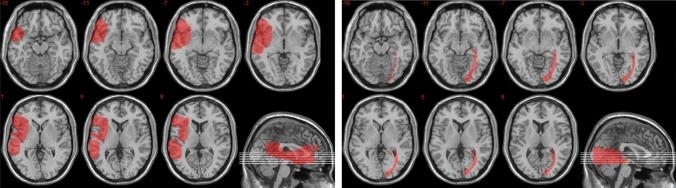
Location of MR’s (left panel) and AT (right panel) brain lesions projected on the mesial view of the standard Montreal Neurological Institute brain. The level of the axial slices is indicated by white horizontal lines on the mesial view of the brain, and by *z*-coordinates. In axial slices, the left hemisphere is on the left side

*Control cases.* MR is a 62-year-old right-handed man with 8 years of education, unemployed, who suffered from a stroke of the right middle cerebral artery. As a result, he has a fronto-temporo-parietal lesion and shows hemispatial neglect (see Table 1 for neuropsychological data and Fig. 2 for lesion mapping). AT is a 65-year-old right-handed man with 8 years of education, retired from work, who suffered from a stroke of the right posterior cerebral artery. As a result, AT has a temporo-occipital lesion, and shows left hemianopsia (see Fig. 2). Because MR and AT are about 10 years older than SG, their performance was compared to that of 10 different healthy controls (all males) matched to them for age and education (mean age = 60.4, range = 58–66, mean education = 10, range = 5–13; *p* > 0.051 in all cases). Participants gave informed consent according to the Declaration of Helsinki (International Committee of Medical Journal Editors 1991) and the Ethical Committee of the Department of Psychology, University of Bologna.

The three cases (SG, AT, and MR) were compared to their healthy controls using individual modified *t*-tests based on Crawford and Garthwaite’s (2002) method for comparing single cases with small control samples. Statistical significance was set at *p* < 0.05, one-tailed.

## Neuropsychological profile

Table 1 shows the neuropsychological profile of SG, MR, and AT. As is apparent from Table 1, all patients showed generally preserved intellectual skills, as revealed by normal performance on the Mini Mental State Examination (Folstein et al. 1975), the Raven Progressive Matrices, and the Verbal Judgment test (for normative data, see Spinnler and Tognoni 1987). All patients had preserved basic attentional skills, as attested by the normal scores they attained at the “Alertness” subtest from the Test battery for Attentional Performance (TAP; for normative data, see Zimmermann and Fimm 2002), and on the Attentional matrices test (Spinnler and Tognoni 1987).

SG and AT performed well also in the “Divided attention” subtest from the TAP, whereas MR had reduced divided attention abilities. Executive functions were generally preserved across patients, as revealed by the normal scores in the Stroop test (Spinnler and Tognoni 1987), the Tower of London test (Culbertson and Zillmer 1999), and verbal fluency measures (Spinnler and Tognoni 1987). SG and AT also showed a preserved performance in the “Go-NoGo” subtests of the TAP, whereas again MR showed a deficit in cognitive control (Zimmermann and Fimm 2002). Short-term memory for both verbal and nonverbal material was within the normal range across patients (Spinnler and Tognoni 1987), whereas working memory, as assessed with the “Working Memory” subtest of TAP (Zimmermann and Fimm 2002), was preserved in SG, but weak in AT and MR. As for long-term memory, all patients had preserved spatial long-term memory, as assessed with the Corsi supra-span test (Spinnler and Tognoni 1987), and verbal long-term memory in a prose recall task, though SG’s performance fell just within the normal limits (Spinnler and Tognoni 1987).

## Experimental investigation

### Task procedure

A set of 180 adjectives reflecting psychological traits was selected from Anderson’s (1968) list and translated to Italian. Ninety adjectives were used in the initial rating phase and served as studied items in the following recognition phase, whereas the remaining 90 adjectives served as distractors in the recognition phase. The assignment of trait adjectives to the different rating conditions or to the distractor status (in the recognition phase) was counterbalanced across participants. In the rating (encoding) phase, participants were presented with 90 adjectives, and required to make different types of judgment depending on the experimental condition, namely, assess whether the adjective described their current psychological traits (Present-Self condition; 18 items), their anticipated psychological traits in 10 years (Future-Self condition; 18 items), the current psychological traits of Gerry Scotti, a famous Italian showman of approximately the same age of our participants (Present-Other condition; 18 items), and the anticipated psychological traits of Gerry Scotti in 10 years (Future-Other condition; 18 items). We also included a Standard condition (18 items), in which participants judged whether the adjective referred to a positive or negative psychological trait, which involves semantic processing but not reflecting on the characteristics of a particular person (self vs. other).

Each trial started with a fixation cross shown for 500 ms. Then a trait adjective appeared, along with the question pertaining to the relevant rating condition (e.g., in the Present-Self condition: how well does this trait describe YOU NOW?), which was written right above the adjective, and remained on the screen until the end of the trial. Across conditions, participants responded using a Likert scale from 1 (not at all) to 4 (totally), with no time limit for responding. Participants evaluated different adjectives in each rating condition (counterbalanced), and the order of trials pertaining to the different conditions was randomized for each participant.

**Fig. 3:**
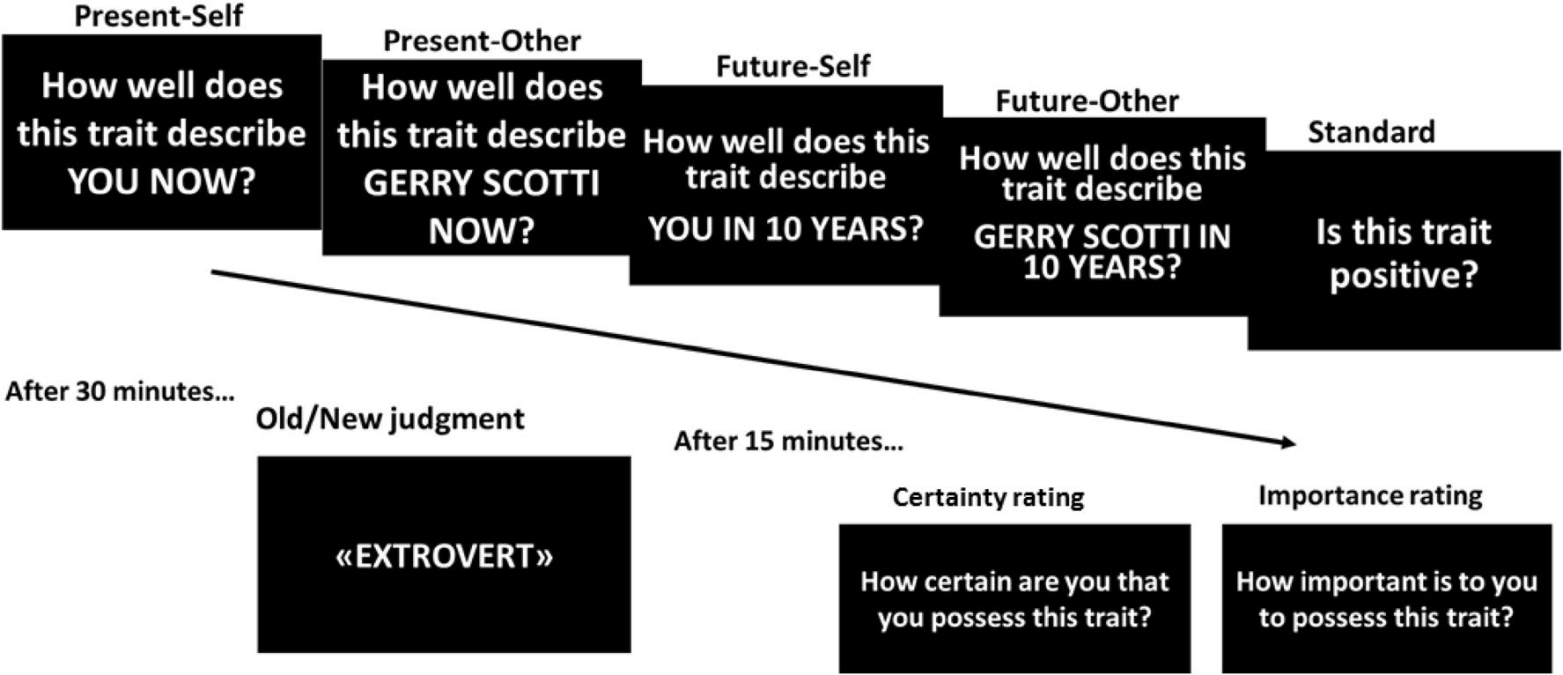
Experimental procedure

About 15 min after the rating phase, which were filled with demographic questionnaires, participants underwent an unanticipated recognition memory task (recognition phase), in which the 90 previously rated adjectives were presented again, this time intermixed with 90 new trait adjectives. Each trial started with a fixation cross shown for 500 ms. Then subjects were presented with an adjective and had to state whether they remembered it from the previous session or not (old/new judgment). Finally, subjects were presented again with the trait adjectives they had previously evaluated with reference to the present or future self (i.e., in the Present-Self and Future-Self conditions of the rating phase; 36 items), and asked to report, for each trait, how sure they were that they possessed or did not possess that trait (epistemic judgment), and how important it was to them to possess or not possess that trait (emotional judgment). In both cases, participants responded using a Likert scale from 1 (not at all) to 4 (totally; see Fig. 3).

## Results

### Rating (encoding) phase

Table 2 shows the mean trait ratings by participant (or participant group) and encoding condition. We first investigated whether there were differences in the degree to which participants attributed psychological traits to the self (Present-self and Future-self conditions), to another person (Present-other and Future-other conditions) or felt that a personality trait was positive (Standard condition). We found no significant differences between SG’s mean ratings and those of his controls, across conditions (*p* > 0.17 in all cases) except for the standard condition, in which SG rated adjectives as generally less positive than his controls (*t* = − 2.4; *p* = 0.02). AT found trait adjectives to be less descriptive of his future self than the controls (*t* = − 3.2; *p* = 0.005), but his ratings in the other conditions were not different from the controls (*p* > 0.16 in all cases). MR found trait adjectives to be more descriptive of the other than the controls (*t* = 1.87; *p* = 0.047), but his ratings in the other conditions were not different from the controls (*p* > 0.059 in all cases).

**Table 2:** Mean trait ratings by participant/participant group and encoding condition. Values in parenthesis are standard deviations

	SG	SG’s controls	AT	MR	AT-MR’s controls
Present Self	2.78	2.62 (0.17)	2.72	2.34	2.74 (0.29)
Future Self	2.39	2.79 (0.30)	2.39	2.56	2.76 (0.11)
Present Other	2.56	2.58 (0.25)	2.95	3.22	2.61 (0.31)
Future Other	2.28	2.42 (0.28)	2.89	3.00	2.61 (0.27)
Standard	2.50	2.78 (0.11)	2.73	2.72	2.88 (0.23)

### Recognition phase

Table 3 shows mean accuracy (hit rates − false alarm rates) by participant (or participant group) and rating condition (Present-self, Future-self, Present-other, Future-other, Standard), and Fig. 2 shows the self-reference effect (SRE) relative to the Present and Future time in SG, AT, MR, and their control groups.

**Table 3:** Mean accuracy (hit rates − false alarm rates) by participant (or participant group) and encoding condition. Values in parenthesis are standard deviations

	SG	SG’s controls	AT	MR	AT-MR’s Controls
Present Self	0.04	0.54 (0.15)	0.10	− 0.02	0.46 (0.18)
Future Self	− 0.12	0.45 (0.12)	0.32	− 0.08	0.44 (0.23)
Present Other	0.10	0.26 (0.15)	− 0.12	− 0.19	0.29 (0.11)
Future Other	0.04	0.27 (0.13)	− 0.01	− 0.13	0.24 (0.21)
Standard	− 0.01	0.44 (0.17)	− 0.01	− 0.02	0.42 (0.14)

Values in parenthesis are standard deviations

#### Standard recognition accuracy

As a preliminary assessment of general recognition memory abilities across participant groups, we compared patients’ recognition accuracy in the Standard condition with that of their controls. As expected, SG (*t* = − 2.52; *p* = 0.02), AT (*t* = − 2.92; *p* = 0.01), and MR (*t* = − 2.99; *p* = 0.008) all had weakened recognition accuracy compared to healthy controls (see Table 3).

#### Recognition accuracy for self and other present and future traits

We next investigated the effect of self-reference and of time on recognition accuracy. As is apparent from Table 3, compared to his controls, SG had a comparable recognition accuracy in the Present Other (*t* = − 1.01; *p* = 0.16) and Future Other conditions (*t* = − 1.68; *p* = 0.06), but a significantly lower recognition accuracy in the present Self (*t* = − 3.17; *p* = 0.006) and Future Self conditions (*t* = − 4.52; *p* = 0.001), indicating he did not benefit from self-referencing in recognition memory. To quantify the SRE directly, we computed Present and Future SRE indices, by subtracting accuracy in the other condition from that in the Self condition, separately for the Present and Future time. We confirmed that both the Present SRE (*t* = − 1.90; *p* = 0.04) and the Future SRE (*t* = − 2.45; *p* = 0.02) were significantly reduced in SG compared to his controls (see Fig. 2).

**Fig. 4:**
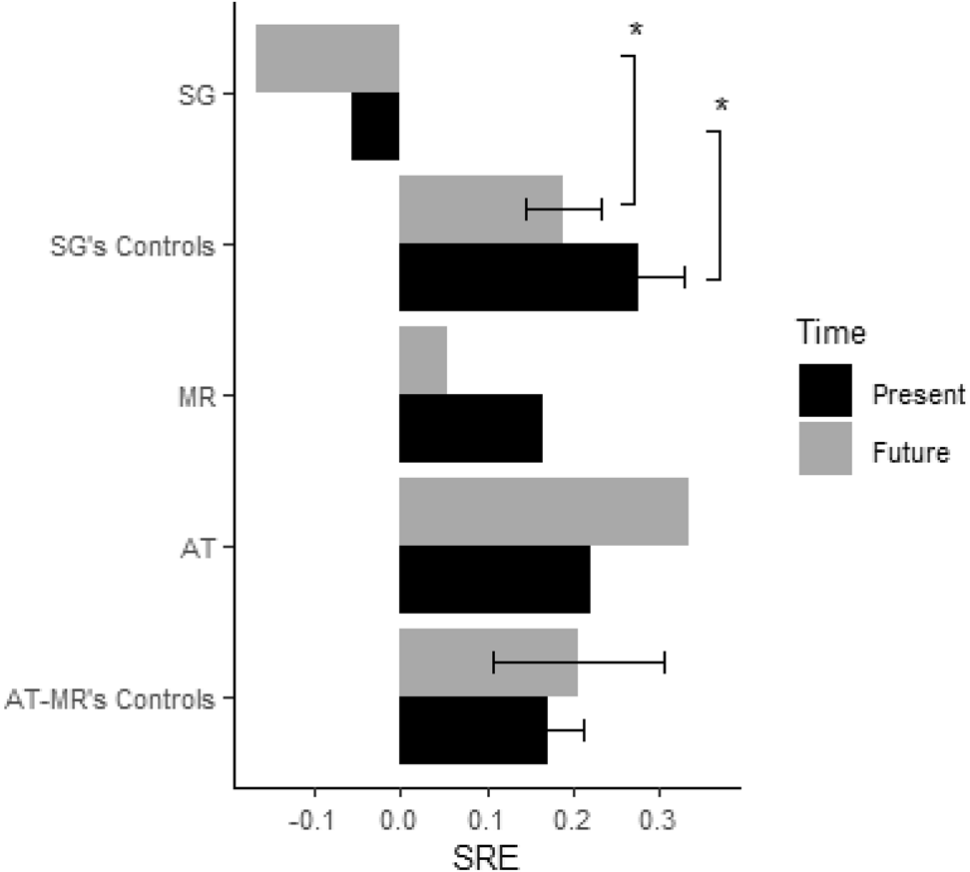
Mean SRE by participant (or participant group) and time condition. Bars represent standard errors; *p < 0.05

The control patients evinced a strikingly different pattern of results. Compared to his controls, MR had a lower recognition accuracy in the Present Other (*t* = − 3.50; *p* = 0.007), Present Self (*t* = − 2.54; *p* = 0.02) and Future Self conditions (*t* = − 2.16; *p* = 0.03), but a comparable recognition accuracy in the Future Other condition (*p* = 0.06). His Present SRE (*p* = 0.50) and Future SRE (*p* = 0.33), however, were comparable to the controls’. AT had a lower recognition accuracy in the Present Self (*t* = − 1.90; *p* = 0.04) and Present Other conditions (*t* = − 2.60; *p* = 0.02) but a comparable recognition accuracy in the Future Self and Future Other conditions (*p* > 0.14 in both cases). His Present SRE (*p* = 0.36) and Future SRE (*p* = 0.31), however, were comparable to the controls’ (see Fig. 4).

This first set of findings shows that the SRE was significantly reduced in SG compared to the controls. In contrast, brain-damaged control patients showed a normal SRE despite their weak baseline recognition memory abilities (Other conditions), indicating they benefited normally from self-referential encoding during a recognition memory task.

### Certainty and importance ratings

To begin to investigate possible reasons for the lack of SRE in SG, we analysed the certainty and importance ratings he and the controls gave to personality traits (see Table 4). SG was less certain to possess (or not) given psychological traits now (Present-self condition; *t* = − 2.11; *p* = 0.03), but his certainty about his personality traits in the Future (*p* = 0.44), as well as importance ratings (both ps > 0.08), did not differ from the controls’. AT gave certainty and importance ratings similar to the controls’ across conditions (*p* > 0.068 in all cases). MR was less certain about (*t* = − 2.17; *p* = 0.03) and attributed less importance (*t* = − 2.50; *p* = 0.01) to possessing (or not) given psychological traits in the Present, but his certainty and importance ratings concerning the Future were similar to the controls’ (*p* > 0.34 in both cases).

**Table 4:** Mean certainty and importance ratings by participant (or participant group) and time condition

Rating	Time	SG	SG’s Controls	AT	MR	AT-MR’s Controls
Certainty	Present	2.50	3.12 (0.28)	3.56	2.56	3.13 (0.25)
	Future	2.89	2.94 (0.35)	3.11	2.59	2.89 (0.44)
Importance	Present	2.62	3.09 (0.45)	3.06	2.56	3.19 (0.24)
	Future	2.67	3.05 (0.40)	3.06	3.00	3.19 (0.50)

Values in parenthesis are standard deviations

## Discussion

In this study, we sought to investigate the integrity of the self-schema in a patient with FRA, S.G. (De Luca et al. 2018), by exploiting the Self-Reference-Effect (SRE), that is, the mnemonic advantage for self- over other-related information. Moreover, we investigated whether retrograde amnesia preclude the anticipation of future self-related information.

To this aim, we contrasted SG’s standard recognition memory and recognition memory for self- and other-related information to that of healthy controls and of two control brain-damaged patients, AT and MR. Compared to healthy controls, all three patients had weak standard recognition memory abilities. However, whereas AT and MR showed significantly higher recognition memory for self-related compared to other-related (personality trait) information, exhibiting a normal SRE, SG did not show any mnemonic advantage for self- over other-related traits. SG’s SRE deficit extended from knowledge about the characteristics of the present self to those anticipated for the future self, leading to a complete abolishment of both the present and future SRE. The lack of SRE in SG was accompanied by a reduced certainty about his current personality traits. Together, the findings of reduced SRE and reduced certainty for his own personality traits in SG (but not the control cases) are suggestive of a disruption of the self-schema following FRA, and not to general encoding deficits following brain damage.

The evidence of reduced self-knowledge in SG accords with the previous finding of his selective deficit in imagining self-relevant future scenarios but not fictitious scenarios with no personal reference (De Luca et al. 2018), and with his tendency to feel pre-morbid acquaintances, passions, and activities as no longer (self-)relevant. Our finding of reduced SRE in association with FRA suggests a link between retrograde memory and self-related knowledge: personal memories feed the self-schema, providing us with a sense of who we are, and serving as the basis to infer how we will likely be like in the future (see also Stendardi et al. 2021). We note that a similar lack of certainty about personality traits was observed also in patient MR, who nonetheless showed a normal SRE. MR’s brain lesion, however, comprises the insula, which is known to be involved in self-reflection and introspection (Picard 2013; van der Meer et al. 2013; Gerretsen et al. 2014; Bartolomei et al. 2019; Sellitto et al. 2016). It is possible, therefore, that insula damage per se reduced (explicit) self-awareness of personality traits in this patient, while leaving the self-schema unaffected.

The association between retrograde amnesia and SRE aligns with previous evidence of a relation between autobiographical memory and self-knowledge, with some authors even holding the strong view that people reminisce and self-reflect on autobiographical memories to build or maintain a stable sense of self and self-continuity (Brewer 1986; Bluck and Levine 1998; Bluck 2003; Bluck and Alea 2008; Wolf and Zimprich 2016). Charlesworth et al. (2016), for example, had participants write a description of a personally relevant event (experimental group) or of a control topic (control group). Then, participants were given 60 s to generate as many self-defining statements as they could, each beginning with “*I am*”. The experimental group was found to produce more self-defining statements compared to the control group, suggesting that retrograde memory boosted self-reflection. Sawczak et al. (2022) found that young healthy participants rated themselves as more empathetic when cued with episodes in which they behaved as empathetic, an effect that was reduced in participants with weaker episodic memory capacity (older adults and patients with temporal lobes damage). Interestingly, Klein and colleagues found that although performing trait judgments did not generally prime episodes in which participants displayed the trait in question (Klein and Loftus 1993; Klein et al. 2002a, b), trait-consistent episodes were found to be consulted while self-reflecting on “ambiguous” traits, that is, those people were not certain to possess, and therefore, no established (semanticized) summary for that trait existed in memory (Klein et al. 1993; Babey et al. 1998; Klein and Lax 2010). These findings suggest that episodic (retrograde) memory can indeed contribute to shaping our sense of self, for example providing the self-schema including multiple, specific instances of our past behavior.

Consistent with this, a globally weaker sense of self has also been reported in patients known to suffer from autobiographical memory impairment, such as patients with Alzheimer’s disease or temporal lobe epilepsy (Rose Addis and Tippett 2004; Penfield and Milner 1958; Ciaramelli et al. 2006; St-Laurent et al. 2009, 2014). Robinson et al. (2017), for example, reported a significant correlation between the quantity of self-attributed personality traits produced by typically developing adolescents and the amount of details in episodic autobiographical memories linked to those traits, a relation that was abolished in young people with autism spectrum disorder, who are known to display a diminished psychological self-knowledge (Lind 2010). As well, Bennouna-Greene et al. (2012) found that in schizophrenic patients autobiographical memories are less connected with self-defining statements than they are in healthy controls.

Consistent with our findings, previous research has shown that self-related knowledge and autobiographical memory share neural bases. They are both associated with activity in the medial prefrontal cortex, precuneus and posterior cingulate cortex (Johnson et al. 2002; Schmitz et al. 2004, 2006; D’Argembeau et al. 2010; Whitfield-Gabrieli et al. 2011), which are part of the default mode network (Buckner et al. 2008; Spreng et al. 2009; Spreng and Grady 2010; Ciaramelli and di Pellegrino 2011; Philippi et al. 2015; Bertossi et al. 2017), even though autobiographical memory retrieval additionally recruits regions associated with visuo-spatial processing, such as the precuneus and inferior parietal lobule (Zysset et al. 2002, 2003; Sajonz et al. 2010). It is worth mentioning that (at a lower threshold) SG displays damage in several regions of the default mode network, including the hippocampus and posterior cingulate cortex, which is the most likely cause of his FRA and lack of SRE (De Luca et al. 2018). The posterior cingulate cortex is well-known for its role in self-reflection and self-referential processing, being consistently engaged in tasks requiring self-trait judgments, self-reflection and self-evaluation (Johnson et al. 2006; D’Argembeau et al. 2008, 2010; Brewer et al. 2013; Martial et al. 2018). The reduced volume in the posterior cingulate cortex might contribute to explain the abnormalities displayed by SG in self-referential processing (present work) and in self-referential future thinking (in De Luca et al. 2018). Of most relevance in this context, the posterior cingulate cortex has been also consistently shown to be involved in autobiographical memory retrieval (Maddock et al. 2001; Svoboda et al. 2006; Summerfield et al. 2009; Sajonz et al. 2010; Bauer et al. 2017), and therefore, may serve as the connective hub of self-knowledge and autobiographical memory.

To conclude, we have provided evidence for a degradation of the self-schema in a case of FRA. This deficit resulted in reduced self-related (trait) knowledge, under-confidence on one’s own personality traits, and inability to envision our self in the future. This deficit is not merely the result of acquired brain damage and (anterograde) amnesia, and reinforce the view of a tight link between (retrograde) autobiographical memory and the sense of self. Together, these findings contribute to specify the cognitive components of mental time travel, and to enrich our understanding of the neuropsychology of FRA.

## Data Availability

Data that support the findings of this study are available upon reasonable request.
